# Supplementary material for: From sequence to scaffold: computational design of protein nanoparticle vaccines from AlphaFold2-predicted building blocks
Source: bioRxiv. 2025 Aug 20:2025.08.20.671178. Preprint. [Version 1] doi: 10.1101/2025.08.20.671178 (PMC12393622; doi:10.1101/2025.08.20.671178)
Supplement: Supplement 1 [file media-1.pdf]

Supporting Information for

From sequence to scaffold: computational design of protein nanoparticle vaccines from AlphaFold2-predicted building blocks

Cyrus M. Haas[a,b,c,1], Naveen Jasti[b,d,1], Annie Dosey[b,e], Joel D. Allen[f], Rebecca Gillespie[g], Jackson McGowan[b,e], Elizabeth M. Leaf[b,e], Max Crispin[f], Cole A. DeForest[a,b,c,h,i,j], Masaru Kanekiyo[g], Neil P. King[b,e,*]

[a]Department of Chemical Engineering, University of Washington, Seattle, WA 98195, USA
[b]Institute for Protein Design, University of Washington, Seattle, WA 98195, USA
[c]Institute for Stem Cell and Regenerative Medicine, University of Washington, Seattle, WA 98195
[d]Department of Molecular Engineering, University of Washington, Seattle, WA 98195, USA
[e]Department of Biochemistry, University of Washington, Seattle, WA 98195, USA
[f]School of Biological Sciences, University of Southampton, Southampton SO17 1BJ, UK
[g]Vaccine Research Center, National Institute of Allergy and Infectious Diseases, National Institutes of Health, Bethesda, MD, 20892
[h]Department of Bioengineering, University of Washington, Seattle, WA 98195, USA
[i]Department of Chemistry, University of Washington, Seattle, WA 98195, USA
[j]Molecular Engineering & Sciences Institute, University of Washington, Seattle, WA 98195, USA

[1]C.M.H. and N.J. contributed equally to this work.
*Corresponding author: neilking@uw.edu

This PDF file includes:

SI Figures S1 to S8
SI Tables S1 to S3
SI References

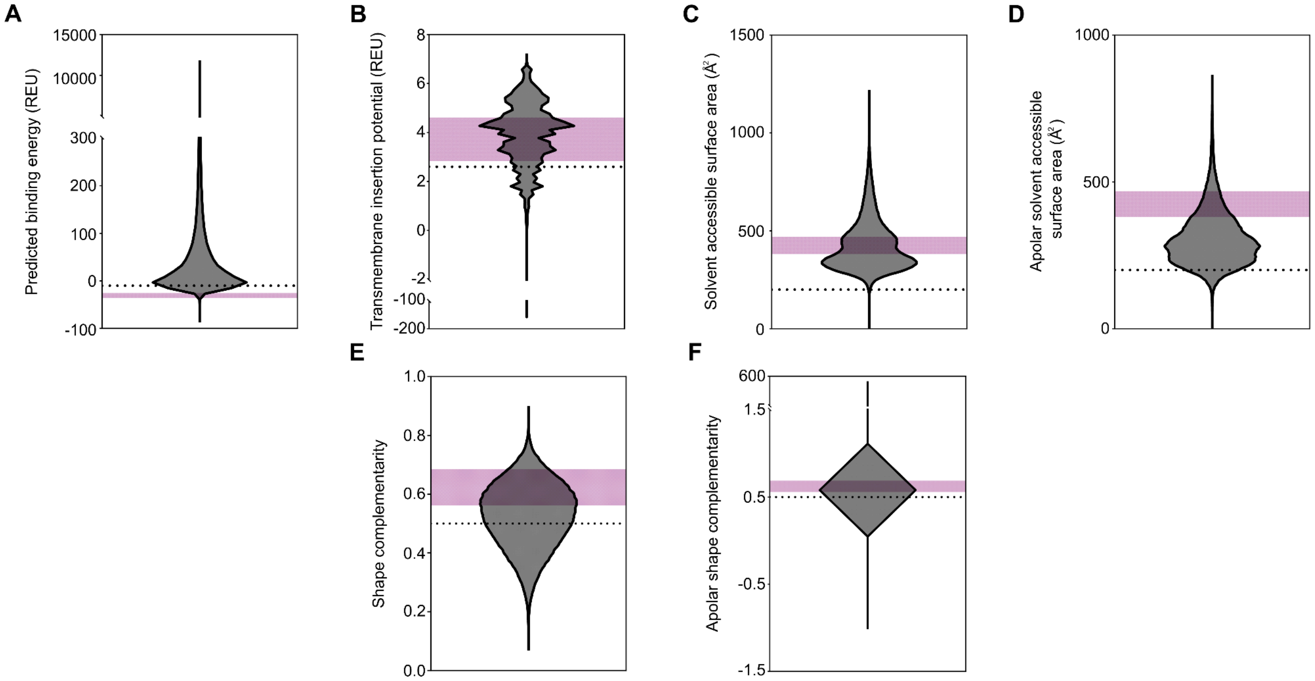

Fig. S1. Summary of Rosetta scoring metrics used to filter and select final nanoparticle designs.
The distribution of data points for the metrics used to filter all designs: *(A)* predicted binding energy (ddG), *(B)* transmembrane insertion potential, *(C)* buried solvent accessible surface area, *(D)* buried apolar solvent accessible surface area, *(E)* shape complementarity, and *(F)* apolar shape complementarity. A small number of values less than zero for buried solvent accessible surface area were attributed to Rosetta calculation errors and are not shown. The dotted lines represent cutoffs used and the magenta regions identify the ranges within which the successfully assembling nanoparticles were selected from.

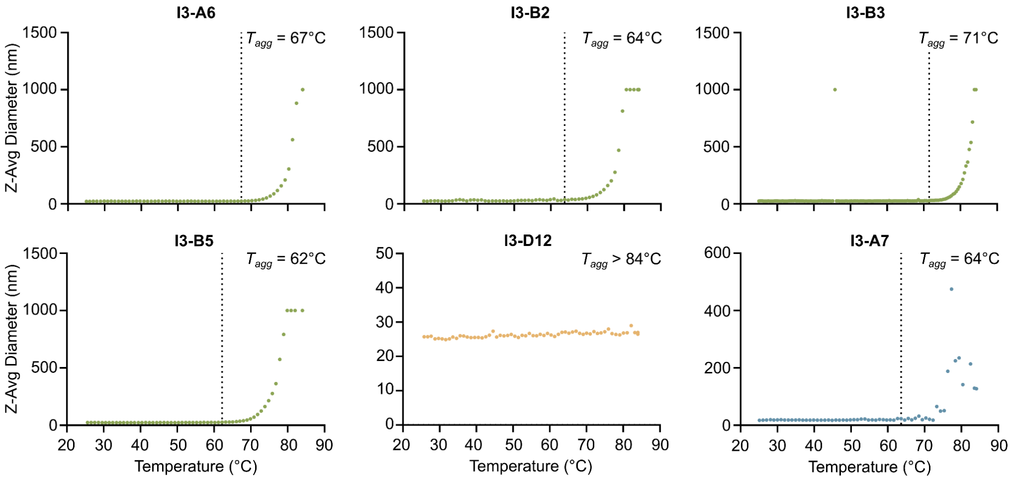

Fig. S2. DLS melting curves used to determine aggregation temperature of nanoparticles. The Z-average diameter of each nanoparticle as determined by DLS with a thermal ramp.

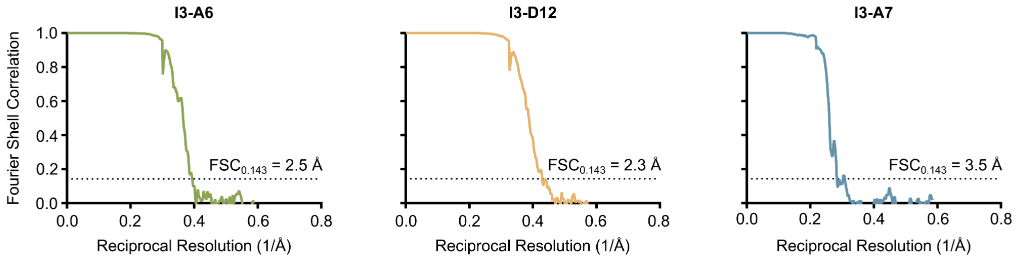

Fig. S3. Cryo-EM gold-standard Fourier shell correlation (FSC) curves.
Gold-standard FSC curves for cryo-EM nanoparticle reconstructions. The 0.143 cutoff is indicated by the dashed line.

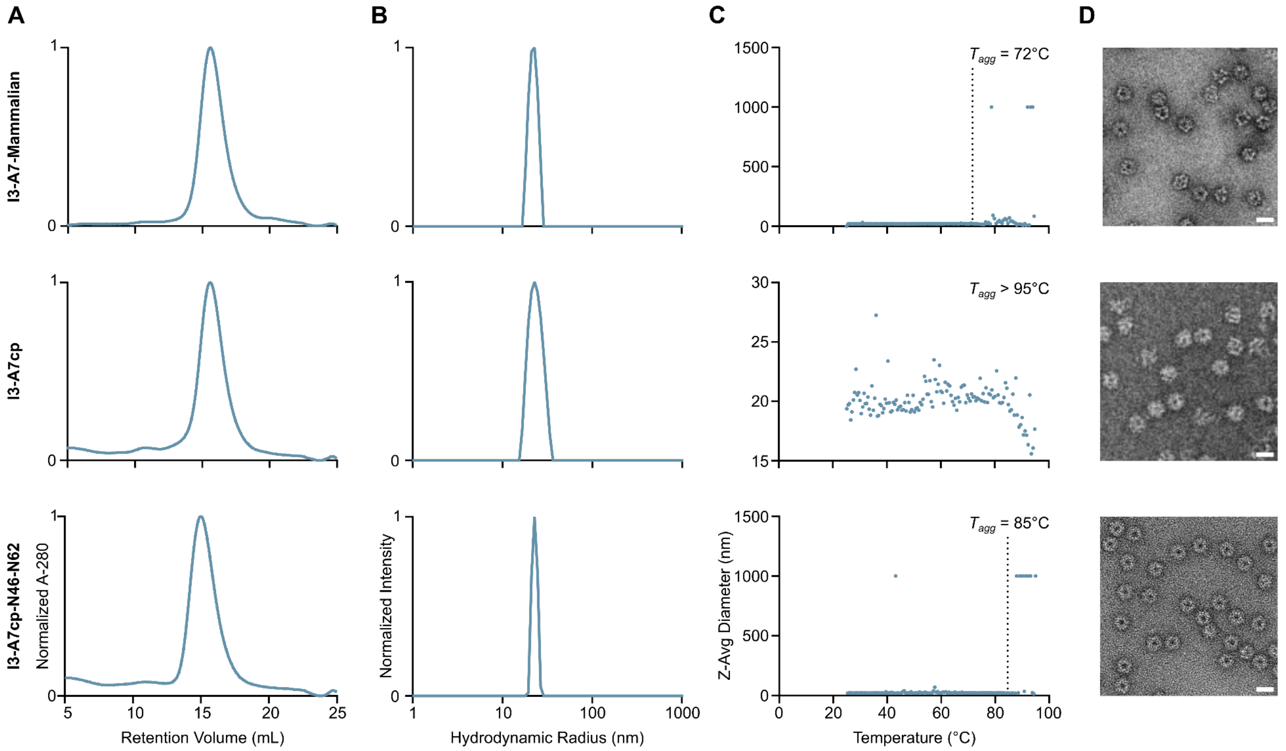

Fig. S4. Characterization of each variant of nanoparticle I3-A7.

(A) SEC traces, *(B)* DLS, *(C)* thermal melting curves, and *(D)* negative stain electron micrographs for I3-A7 secreted from mammalian cells, the circular permuted version (I3-A7cp), and the permutation with the two N-linked glycans at N46 and N62. Micrographs for I3-A7cp and I3-A7cp-N46-N62 are differently cropped images of those shown in Figure 4. Scale bar = 21 nm.

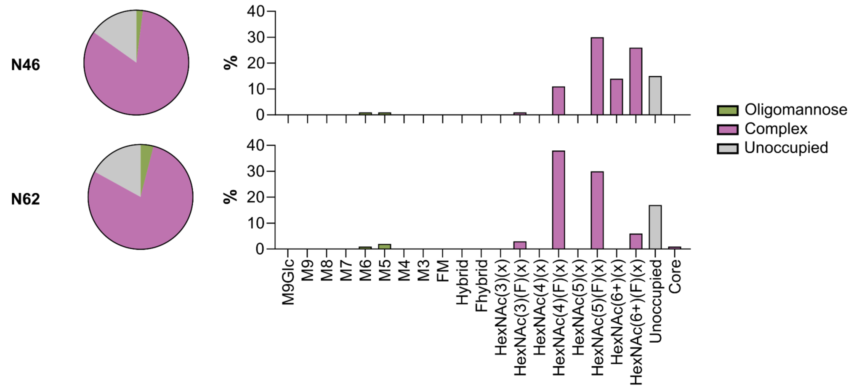

Fig. S5. Characterization of glycans N46 and N62 in the I3-A7cp-N46-N62-NonAssembling trimer.
The glycan composition of peptides containing the N46 (top) and N62 (bottom) glycosylation sites of the I3-A7cp-N46-N62-NonAssembling trimer determined by LC-MS.

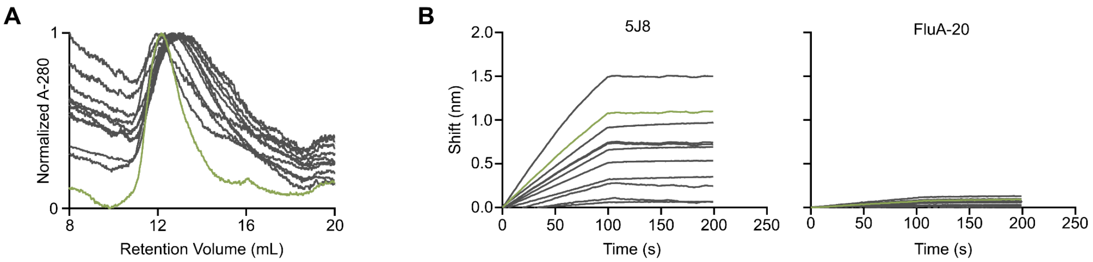

Fig. S6. Experimental characterization of trihead-bearing I3-A7cp nanoparticles with eleven diffused linkers by SEC and BLI.

(A) SEC traces of the eleven potential vaccine candidates. *(B)* BLI of nanoparticle vaccine candidates binding to 5J8 and FluA-20. The green line in all plots signifies the final candidate tested *in vivo* (TH-I3-A7).

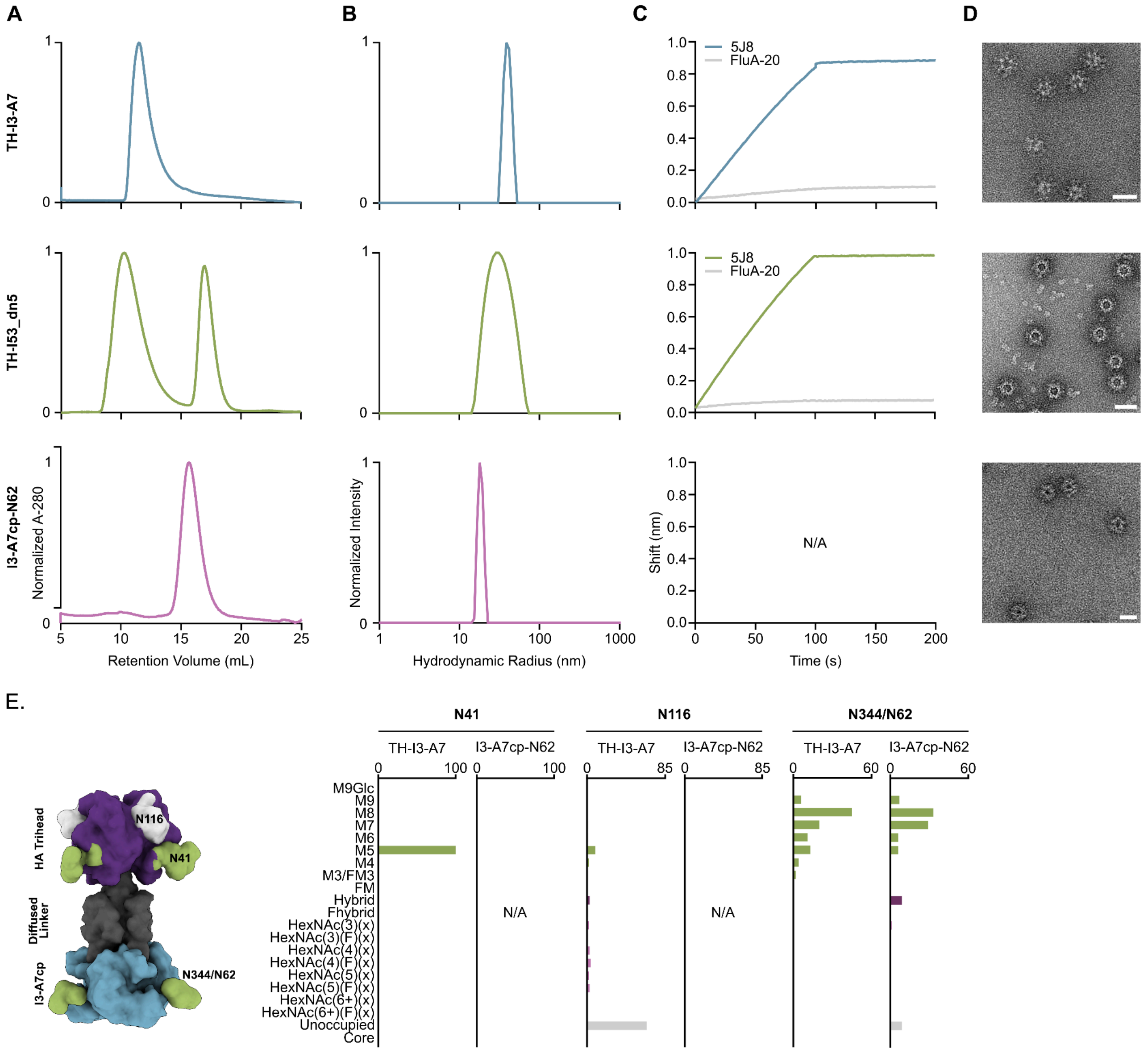

Fig. S7. Biochemical, biophysical, and antigenic characterization of nanoparticle immunogens.
(A) SEC traces, *(B)* DLS, *(C)* BLI, and *(D)* negative stain EM of the three immunogens used in the mouse immunogenicity study shown in Figure 5. Scale bar = 40 nm, 42 nm, and 21 nm for TH-I3-A7, TH-I53_dn5, and I3-A7cp-N62, respectively. *(E)* Characterization of the three primary glycosylation sites on TH-I3-A7. N41 and N116 are in the trihead antigen, and N344/N62 is in I3-A7cp. BLI and negative stain EM for TH-I3-A7 are shown here in addition to Figure 5 for comparison.

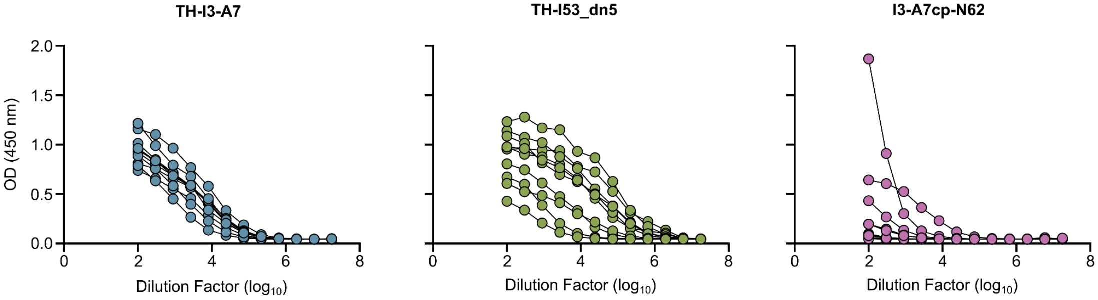

Fig. S8. ELISA curves for mouse serum antibody binding to a full-length MI15 ectodomain trimer.
Week six serum ELISA curves against a vaccine-matched full-length HA ectodomain trimer (A/Michigan/45/2015).

Table S1. Interface Mutations of Designed Nanoparticles

Nanoparticle	PDB Seed	Homolog Source	Homolog Sequence	Nanoparticle Sequence
I3-A6	6HS8	*Clostridium sp.*<br>CAG:307_30_263;<br>Genbank Accession<br>OKZ90856	MKILVINGPNINFLGIREKG<br>IYGNDNYDTLVDMINKKAE<br>ELNVKVEVFQSNHEGAIID<br>KLQEAYYNDVDGIVINPGA<br>FTHYSYAVRDALASIAAIP<br>KIEVHISNVHTREEFRHTS<br>VTVPVCQGQVVGLGLRG<br>YLYAMEALTDMTKSN	MKILVINGPNINFLGIREKG<br>IYG**PL**NYD**D**LV**E**MI**KGT**A**K**<br>**G**L**K**VKVEVFQSNHEGAIID<br>KLQEAYYNDVDGIVINPGA<br>FTHYSYAVRDALASIAAIP<br>KIEVHISNVHTREEFRHTS<br>VTVPVCQG**E**VVGLGL**G**G<br>YL**A**AM**GM**L**VE**MTKSN
I3-A7	6HF7	*Thermoplasmata archaeon HGW-Thermoplasmata -1*; Genbank Accession<br>PKK86397	MGAIVVTGIPGVGKTTVM<br>QKAAEGMNIEFVTYGTEM<br>FEVAKSMGLVKDRDEMR<br>RLSPDVQKEVQKKAAENI<br>AAKGNVILDTHCTIKTPKG<br>YLPGLPRWVLEKLRPSVIL<br>LVEADPKEIYGRRLKDET<br>RNRDPDSEEEIAEHQMM<br>NRAAAMAYASLSGATVKIV<br>FNHDNRLDDAVRDAAPVL<br>GN	MGAIVVTGIPGVGKTTVM<br>QKAAEG**SPLPR**V**PLEGV**<br>M**YG**VAK**R**MGLVKD**I**DEMR<br>RLSPDVQKEVQKKAAE**R**I<br>AA**L**G**D**VILDTHCTIKTPKG<br>YLPGLPRWVLEKLRPSVIL<br>LVEADPKEIYGRRLKDET<br>RNRDPDSEEEIAEHQMM<br>NRAAAMAYASLSGATVKIV<br>FNHDNRLDDAVRDAAPVL<br>GN
I3-B2	6HS8	*Clostridium sp.*<br>CAG:307_30_263;<br>Genbank Accession<br>OKZ90856	MKILVINGPNINFLGIREKG<br>IYGNDNYDTLVDMINKKAE<br>ELNVKVEVFQSNHEGAIID<br>KLQEAYYNDVDGIVINPGA<br>FTHYSYAVRDALASIAAIP<br>KIEVHISNVHTREEFRHTS<br>VTVPVCQGQVVGLGLRG<br>YLYAMEALTDMTKSN	MKILVINGPNINFLGIREKG<br>IYG**KY**NYD**F**LVD**L**IN**ET**A**K**<br>**L**LNVKVEVFQSNHEGAIID<br>KLQEAYYNDVDGIVINPGA<br>FTHYSYAVRDALASIAAIP<br>KIEVHISNVHTREEFRHTS<br>VTVPVCQG**EID**G**A**GL**G**GY<br>**VS**AM**LT**L**VE**MTKSN
I3-B3	6HS8	*Clostridium sp.*<br>CAG:307_30_263;<br>Genbank Accession<br>OKZ90856	MKILVINGPNINFLGIREKG<br>IYGNDNYDTLVDMINKKAE<br>ELNVKVEVFQSNHEGAIID<br>KLQEAYYNDVDGIVINPGA<br>FTHYSYAVRDALASIAAIP<br>KIEVHISNVHTREEFRHTS<br>VTVPVCQGQVVGLGLRG<br>YLYAMEALTDMTKSN	MKILVINGPNINFLGIREKG<br>IYG**PF**NYD**F**LV**EL**INK**T**AE<br>**L**LNVKVEVFQSNHEGAIID<br>KLQEAYYNDVDGIVINPGA<br>FTHYSYAVRDALASIAAIP<br>KIEVHISNVHTREEFRHTS<br>VTVPVCQG**E**V**D**G**A**GL**G**G<br>Y**VS**AME**R**L**VS**MTKSN
I3-B5	6HS8	*Clostridium sp.*<br>CAG:307_30_263;<br>Genbank Accession<br>OKZ90856	MKILVINGPNINFLGIREKG<br>IYGNDNYDTLVDMINKKAE<br>ELNVKVEVFQSNHEGAIID<br>KLQEAYYNDVDGIVINPGA<br>FTHYSYAVRDALASIAAIP<br>KIEVHISNVHTREEFRHTS<br>VTVPVCQGQVVGLGLRG<br>YLYAMEALTDMTKSN	MKILVINGPNINFLGIREKG<br>IYG**PF**NYD**D**LVD**L**INK**T**AE<br>**F**LNVKVEVFQSNHEGAIID<br>KLQEAYYNDVDGIVINPGA<br>FTHYSYAVRDALASIAAIP<br>KIEVHISNVHTREEFRHTS<br>VTVPVCQGQV**D**G**A**GL**A**G<br>Y**VQ**AM**FE**LT**S**MTKSN

I3-D12	1QWG	*Methanocaldococcus jannaschii*; Genbank Accession HII60133	MKAFEFLYEDFQRGLTVV<br>LDKGLPPKFVEDYLKVCG<br>DYIDFVKFGWGTSAVIDR<br>DVVKEKINYYKDWGIKVY<br>PGGTLFEYAYSKGKFDEF<br>LNECEKLGFEAVEISDGS<br>SDISLEERKNAIKRAKDNG<br>FMVLTEVGKKMPDKDKQL<br>TIDDRIKLINFDLDAGADY<br>VIIEGRESGKGIGLFDKEG<br>KVKENELDVLAKNVDINK<br>VIFEAPQKSQQVAFILKFG<br>SSVNLANIAFDEVISLETL<br>RRGLRGDTFGKV	MKAFEFLYEDFQRGLTVV<br>LDKGLPPKFVEDYLKVCG<br>DYIDFVKFGWGTSAVIDR<br>DVVKEKINYYKDWGIKVY<br>PGGTLFEYAYSKG**EAA**EF**I**<br>**A**EC**K**KLGFEAVEISDGSS<br>DISL**DD**RK**A**AI**WG**AK**WA**G<br>FMVLTEVGKKMPDKDKQL<br>TIDDRIKLINFDLDAGADY<br>VIIEGRESGKGIGLFDKEG<br>KVKENELDVLAKNVDINK<br>VIFEAPQKSQQVAFILKFG<br>SSVNLANIAFDEVISLETL<br>RRGLRGDTFGKV

Bolded and underlined letters in the nanoparticle sequences represent the mutations designed for nanoparticle assembly.

Table S2. Amino Acid Sequences

Construct	Sequence
I3-A6	*MTDEPVATEEPAARDEVRRAEALGGQAAEQLA*MKILVINGPNINFLGIREKGIYGP<br>LNYDDLVEMIKGTAKGLKVKVEVFQSNHEGAIIDKLQEAYYNDVDGIVINPGAFTHY<br>SYAVRDALASIAAIPKIEVHISNVHTREEFRHTSVTVPVCQGEVVGLGLGGYLAAM<br>GMLVEMTKSN**GSWGSLEHHHHHH**
I3-B2	*MTDEPVATEEPAARDEVRRAEALGGQAAEQLA*MKILVINGPNINFLGIREKGIYGK<br>YNYDFLVDLINETAKLLNVKVEVFQSNHEGAIIDKLQEAYYNDVDGIVINPGAFTHY<br>SYAVRDALASIAAIPKIEVHISNVHTREEFRHTSVTVPVCQGEIDGAGLGGYVSAML<br>TLVEMTKSN**GSWGSLEHHHHHH**
I3-B3	*MTDEPVATEEPAARDEVRRAEALGGQAAEQLA*MKILVINGPNINFLGIREKGIYGP<br>FNYDFLVELINKTAELLNVKVEVFQSNHEGAIIDKLQEAYYNDVDGIVINPGAFTHY<br>SYAVRDALASIAAIPKIEVHISNVHTREEFRHTSVTVPVCQGEVDGAGLGGYVSAM<br>ERLVSMTKSN**GSWGSLEHHHHHH**
I3-B5	*MTDEPVATEEPAARDEVRRAEALGGQAAEQLA*MKILVINGPNINFLGIREKGIYGP<br>FNYDDLVDLINKTAEFLNVKVEVFQSNHEGAIIDKLQEAYYNDVDGIVINPGAFTHY<br>SYAVRDALASIAAIPKIEVHISNVHTREEFRHTSVTVPVCQGQVDGAGLAGYVQAM<br>FELTSMTKSN**GSWGSLEHHHHHH**
I3-D12	MKAFEFLYEDFQRGLTVVLDKGLPPKFVEDYLKVCGDYIDFVKFGWGTSAVIDRD<br>VVKEKINYYKDWGIKVYPGGTLFEYAYSKGEAAEFIAECKKLGFEAVEISDGSSDIS<br>LDDRKAAIWGAKWAGFMVLTEVGKKMPDKDKQLTIDDRIKLINFDLDAGADYVIIE<br>GRESGKGIGLFDKEGKVKENELDVLAKNVDINKVIFEAPQKSQQVAFILKFGSSVN<br>LANIAFDEVISLETLRRGLRGDTFGKV**LEHHHHHH**
I3-A7<br>(bacterial expression)	MGAIVVTGIPGVGKTTVMQKAAEGSPLPRVPLEGVMYGVAKRMGLVKDIDEMRR<br>LSPDVQKEVQKKAAERIAALGDVILDTHCTIKTPKGYLPGLPRWVLEKLRPSVILLV<br>EADPKEIYGRRLKDETRNRDPDSEEEIAEHQMMNRAAAMAYASLSGATVKIVFNH<br>DNRLDDAVRDAAPVLGN**LEHHHHHH**
I3-A7<br>(mammalian expression)	(MDSKGSSQKGSRLLLLLVVSNLLLPQGVLA)MGAIVVTGIPGVGKTTVMQKAAEG<br>SPLPRVPLEGVMYGVAKRMGLVKDIDEMRRLSPDVQKEVQKKAAERIAALGDVIL<br>DTHCTIKTPKGYLPGLPRWVLEKLRPSVILLVEADPKEIYGRRLKDETRNRDPDSE<br>EEIAEHQMMNRAAAMAYASLSGATVKIVFNHDNRLDDAVRDAAPVLGN**GSHHHH**<br>**HH**
I3-A7cp	(MWWRLWWLLLLLLLLWPMVWAAA)**HHHHHHGSGSGS**SEEEIEEHQLINRYAAM<br>AYAVLSGATVKIVFNHDNRLDDAVRDAAPVLHEGEGVIVVTGIPGVGKTTVMQKAA<br>EGSPLPRVPLEGVMYGVAKRMGLVKDIDEMRRLSPDVQKEVQKKAAERIAALGD<br>VILDTHCTIKTPKGYLPGLPRWVLEKLRPSVILLVEADPKEIYGRRLKDETRNRDPD
I3-A7cp-N46-N62	(MWWRLWWLLLLLLLLWPMVWAAA)**HHHHHHGSGSGS**SEEEIEEHQLINRYAAM<br>AYAVLSGATVKIVFNHNNTLDDAVRDAAPVLHNGTGVIVVTGIPGVGKTTVMQKAA<br>EGSPLPRVPLEGVMYGVAKRMGLVKDIDEMRRLSPDVQKEVQKKAAERIAALGD<br>VILDTHCTIKTPKGYLPGLPRWVLEKLRPSVILLVEADPKEIYGRRLKDETRNRDPD

I3-A7cp-N111	(MWWRLWWLLLLLLLLWPMVWAAA)**HHHHHHGSGSGS**SEEEIEEHQLINRYAAM<br>AYAVLSGATVKIVFNHDNRLDDAVRDAAPVLHEGEGVIVVTGIPGVGKTTVMQKAA<br>EGSPLPRVPLEGVMYGVAKRMGLVKNITEMRRLSPDVQKEVQKKAAERIAALGD<br>VILDTHCTIKTPKGYLPGLPRWVLEKLRPSVILLVEADPKEIYGRRLKDETRNRDPD
I3-A7cp-N46-N62-NonAssembling	(MWWRLWWLLLLLLLLWPMVWAAA)**HHHHHHGSGSGS**SEEEIEEHQLINRYAAM<br>AYAVLSGATVKIVFNHNNTLDDAVRDAAPVLHNGTGVIVVTGIPGVGKTTVMQKAA<br>EGMNIEFVTYGTEMFEVAKSMGLVKDRDEMRRLSPDVQKEVQKKAAENIAAKGN<br>VILDTHCTIKTPKGYLPGLPRWVLEKLRPSVILLVEADPKEIYGRRLKDETRNRDPD
I3-A7cp-N62	(MWWRLWWLLLLLLLLWPMVWAAA)**HHHHHHGSGSGS**SEEEIEEHQLINRYAAM<br>AYAVLSGATVKIVFNHDNRLDDAVRDAAPVLHNGTGVIVVTGIPGVGKTTVMQKAA<br>EGSPLPRVPLEGVMYGVAKRMGLVKDIDEMRRLSPDVQKEVQKKAAERIAALGD<br>VILDTHCTIKTPKGYLPGLPRWVLEKLRPSVILLVEADPKEIYGRRLKDETRNRDPD
TH-I3-A7	(MDSKGSSQKGSRLLLLLVVSNLLLPQGVLA)VAPLHLGKCNIAGWILGNPECESLS<br>TASSWSYIVETSNSDNGTCFPGNFINYEELRCQLSSVSSFERFEIFPKTSSWPNH<br>DSNKGVTAACPHAGAKSFYKNLIWLVKKGNSYPKLNQSYINDKGKEVLVLWGIHH<br>PSTTADQQSLYQNEDTYVFVSTSRYDKVFKPIIATRPKVRDQEGRMNYYWTLVEP<br>GDKITFEATGNLVVPRYAFTMERNAGSGSGSCIENINSKIYHIENKLALDRLILALEA<br>KEAGKDWEGIEKVIKELEKVAKDDEEAKKFLEKKKKELEEEKEEWKREEEIEEHQ<br>LINRYAAMAYAVLSGAEVKIVFNHDNRLDDAVRDAAPVLHNGTGVIVVTGIPGVGK<br>TTVMQKAAEGSPLPRVPLEGVMYGVAKRMGLVKDIDEMRRLSPDVQKEVQKKAA<br>ERIAALGDVILDTHCTIKTPKGYLPGLPRWVLEKLRPSVILLVEADPKEIYGRRLKD<br>ETRNRDPD**GSGSHHHHHH**
TH-I53_dn5<br>(A component : B component)	MGKYDGSKLRIGILHARGNAEIILELVLGALKRLQEFGVKRENIIIETVPGSFELPYG<br>SKLFVEKQKRLGKPLDAIIPIGVLIRGSTAHFDYIADSTTHQLMKLNFELGIPVIFGVL<br>TTESDEQAEERAGTKAGNHGEDWGAAAVEMATKFN**HHHHHH** :<br>(MDSKGSSQKGSRLLLLLVVSNLLLPQGVLA)VAPLHLGKCNIAGWILGNPECESLS<br>TASSWSYIVETSNSDNGTCFPGNFINYEELRCQLSSVSSFERFEIFPKTSSWPNH<br>DSNKGVTAACPHAGAKSFYKNLIWLVKKGNSYPKLNQSYINDKGKEVLVLWGIHH<br>PSTTADQQSLYQNEDTYVFVSTSRYDKVFKPIIATRPKVRDQEGRMNYYWTLVEP<br>GDKITFEATGNLVVPRYAFTMERNAGSGSGSCIEHIENEIAELAYLLGELAYKLGEY<br>RIAIRAYRIALKSDPNNAEAWYNLGNAYYKQGRYREAIEYYQKALELDPNNAEAW<br>YNLGNAYYERGEYEEAIEYYRKALRLDPNNADAMQNLLNAKMREEGGWELQ**HH<br>HHHH**
Full-length HA ectodomain trimer (A/Michigan/45/2015), including stabilizing mutations from Milder et al. (1)	(MDSKGSSQKGSRLLLLLVVSNLLLPQGVLA)DTLCIGYHANNSTDTVDTVLEKNV<br>TVTHSVNLLEDKHNGKLCKLRGVAPLHLGKCNIAGWILGNPECESLSTASSWSYIV<br>ETSNSDNGTCFPGDFINYEELREQLSSVSSFERFEIFPKTSSWPNHDSNKGVTAA<br>CPHAGAKSFYKNLIWLVKKGNSYPKLNQSYINDKGKEVLVLWGIHHPSTTADQQS<br>LYQNADAYVFVGTSRYSKKFKPEIATRPKVRDQEGRMNYYWTLVEPGDKITFEAT<br>GNLVVPRYAFTMERNAGSGIIISDTPVHDCNTTCQTPEGAINTSLPFQNIHPITIGK<br>CPKYVKSTKLRLATGLRNVPSIQSRGLFGAIAGFIEGGWTGMVDGWYGYHWQNE<br>QGSGYAADLKSTQNAIDKITNIVNSVIEKMNTQFTAVGKEFNHLEKRIENLNKKVDD<br>GFLDIWTYNAELLVLLINERTLDYHDSNVKNLYEKVRNQLKNNAKEIGNGCFEFYH<br>KCDNTCMESVKNGTYDYPKYSEEAKLNREKIDGVGSGYIPEAPRDGQAYVRKDG<br>EWVLLSTFLGSGLNDIFEAQKIEWHEG**HHHHHH**

Signal peptides are indicated in parentheses and purification tags are bolded and underlined.
Italicized and underlined sections of sequences from I3-A6, I3-B2, I3-B3, I3-B5 represent mistakenly added sequence from an unrelated thermophilic organism, *Thermasporomyces composti*, due to an error in BLAST retrieval of full length genomic sequences.
This region was not resolved by cyro-EM and is unlikely to have an impact on nanoparticle assembly.

Table S3. Cryo-EM Data Collection Statistics

	I3-A6<br>PDB: 9CLZ<br>EMD: 45734	I3-D12<br>PDB: 9CM1<br>EMD: 45736	I3-A7<br>PDB: 9CM0<br>EMD: 45735
**Data collection and processing**			
Magnification (×)	105,000	105,000	105,000
Voltage (kV)	300	300	300
Electron exposure ($e^-/Å^2$)	52	52	52
Defocus range (μm)	-0.5 to -2.0	-0.5 to -2.0	-0.5 to -2.0
Pixel Size (Å)	0.843	0.843	0.843
Symmetry imposed	I	I	I
Final particle images (no.)	290,122	321,856	165,931
Map resolution (Å)	2.5	2.3	3.5
FSC threshold	0.143	0.143	0.143
**Validation**			
MolProbity score	1.03	0.53	0.57
Clashscore	0.26	0.08	0.18
Poor rotamers (%)	1.94	0.33	0.7
Ramachandran plot			
Favored (%)	96.68	99.15	97.07
Allowed (%)	100	100	100
Disallowed (%)	0	0	0

SI References

1. F. J. Milder, *et al.*, Universal stabilization of the influenza hemagglutinin by structure-based redesign of the pH switch regions. *Proc. Natl. Acad. Sci. U. S. A.* **119** (2022).
